# Radiation Oncology *In Vitro*: Trends to Improve Radiotherapy through Molecular Targets

**DOI:** 10.1155/2014/461687

**Published:** 2014-09-15

**Authors:** Natália Feofanova, Jony Marques Geraldo, Lídia Maria de Andrade

**Affiliations:** ^1^Research Institute of Internal and Preventive Medicine, FSBI, Boris Bogatkov Street 175/1, Novosibirsk 630089, Russia; ^2^School of Medicine, Federal University of Minas Gerais, Avenida Alfredo Balena, 190 Santa Efigênia, 30130-100 Belo Horizonte, MG State, Brazil; ^3^Alberto Cavalcanti Hospital, Minas Gerais State Hospital Foundation-FHEMIG, Rua Camilo de Brito, 636 Padre Eustáquio, 30730-540 Belo Horizonte, MG, Brazil; ^4^Department of Physics, Institute of Mathematical Sciences, Federal University of Minas Gerais, Avenida Antônio Carlos 6627, 31270-901 Belo Horizonte, MG, Brazil

## Abstract

Much has been investigated to improve the beneficial effects of radiotherapy especially in that case where radioresistant behavior is observed. Beyond simple identification of resistant phenotype the discovery and development of specific molecular targets have demonstrated therapeutic potential in cancer treatment including radiotherapy. Alterations on transduction signaling pathway related with MAPK cascade are the main axis in cancer cellular proliferation even as cell migration and invasiveness in irradiated tumor cell lines; then, for that reason, more studies are in course focusing on, among others, DNA damage enhancement, apoptosis stimulation, and growth factors receptor blockages, showing promising *in vitro* results highlighting molecular targets associated with ionizing radiation as a new radiotherapy strategy to improve clinical outcome. In this review we discuss some of the main molecular targets related with tumor cell proliferation and migration as well as their potential contributions to radiation oncology improvements.

## 1. Introduction

To achieve a better understanding of the different targeted cancer responsiveness a wide range of experimental tumors of various histologic types have been developed for radiobiological studies [1] whose effects, induced by ionizing radiation (IR), can be investigated through many approaches, allowing the identification of radioresistance or radiosensitivity of human cancer cell lines [2]. There are, for instance, similar behaviors between clonogenic repopulation* in vitro* applying fractioned schedule on human squamous cell carcinoma and patients that were treated with radiotherapy [3]. Nevertheless, several target molecules into different subcellular compartments related with radioresistance were already identified as an attempt to improve cellular radiation responses and in another aspect a number of agents targeting components of cell signaling pathways and processes critical to neoplastic transformation and progression are ongoing clinical development [4]. Amongst other signaling cascades targets, there are some related with cell dynamics in an integrin dependent fashion that increases cancer cell migration induced by IR, like beta-galactoside alpha-(2,6)-sialyltransferase (ST6Gal I), that was found overexpressed in ovarian and other cancers whose expression has been correlated to metastasis and poor prognosis [5, 6]. In colon cancer cells IR increases the expression of ST6Gal I, which, in turn, is involved in radioresistance and radiation-induced migration via sialylation of integrin*β*_1_ that may be a novel target for overcoming radiation-induced survival, especially adhesion and migration of this kind of tumor [7, 8]. Another well-known type of molecules involved in cancer progression after IR is the members of a disintegrin and metalloproteinase (ADAM) family that are thought to mediate the shedding of epidermal growth factor receptor (EGFR) ligands, an important signaling pathway to cell proliferation and migration, and this event is critical for a more soluble functional EGFR ligands yield. ADAM is activated by IR leading to an increased triggering cell proliferation cascade in irradiated melanoma cells [9], specifying ADAM's blockage as an attractive target to radiosensitize this tumor type. Another important subject is radio-induced DNA damage and its repair ability that have been largely investigated including the role of tyrosine kinase pathways such as the ataxia-telangiectasia mutated (ATM), a protein kinase that is best known for its role in the DNA damage response [10], and one recent work describes a new function for 5′-adenosine monophosphate- (AMP-) activated protein kinase (AMPK), an established metabolic stress sensor, that has the ability to control cellular growth and mediate cell cycle checkpoints in cancer cells in response to low energy levels, as a sensor of genomic stress and a participant of the DNA damage response (DDR) pathway, highlighting the importance of targeting AMPK as novel cancer therapeutics for a radiosensitization of human cancer cells, mediated by a simultaneous inhibition of the Akt and activation of the AMPK signaling pathways [11–13].

The knowledge in tumor biology has been increased in the last years due to cellular signaling pathways discovering, mechanistically identified by molecular tools that allowed investigation of an enormous range of possibilities, even as new mathematical models have also been subsidizing radiotherapy enhancement. The understanding and identification of specific molecular targets with significant therapeutic implications in order to develop new strategies for radiotherapy are crucial to improve patient survival without substantially increasing toxicity.

## 2. Radiobiological Models

Delivery of advanced radiotherapy techniques has taken new approaches to treatment including stereotactic irradiation and intensity-modulated X-rays beam in order to improve outcomes of cancer treatment and reduce damage to normal surrounding tissues [14]. The classical well-known radiobiological models are linear-quadratic (LQ) and biologically effective dose (BED) that are widely used to estimate the effects of various radiation schedules, but it has been suggested that LQ is not applicable to high doses per fraction [15] due to the fact that LQ overestimates effects of high daily radiation doses proving that better models should be proposed [16]. Thus, different treatment schedules applying hypofractionated radiotherapy (hRT) and other radiation modalities such as light ions have been used with the same proposal, even though the pattern of received dose is different from that in conventional radiation and therefore the radiobiological aspects of cell death are shown to be modified. Prolonged or short radiation delivery makes sublethal damage repair, repopulation, and reoxygenation be better evaluated specially when a mathematical model is used for dose conversion from conventional treatment to high daily hypofractionated doses [17]. For instance, dose conversion models as repairable-conditionally repairable (RCR) model [18] and multitarget (MT) model are currently recommended. When those models were compared LQ seemed to fit relatively well at doses of 5 Gy or less; at 6 Gy or higher doses, RCR and MT models seemed to be more reliable than LQ [19]. In hypofractionated stereotactic radiotherapy, LQ model should not be used, and conversion models incorporating the concept of RCR or MT models, such as generalized linear-quadratic (gLQ) models, appear to be more suitable [20, 21].

Recent investigations have highlighted differential cellular responses when submitted to intensity-modulated radiation fields [22], particularly in areas outside the primary treatment fields [23]. Differential DNA damage responses following modulated radiation field delivery were found, providing an evidence for a role of intercellular communication in mediating cellular radiobiological response to modulated radiation fields, suggesting that advanced radiotherapy treatment plans require a refinement of existing radiobiological modeling [24]. Concerning radiobiology, DNA double strand breaks (DSB) are considered to be the kind of DNA damage responsible for most end points such as chromosome aberrations and cell killing. However, due to high number of DSBs induced by radiation at sublethal doses, it is immediately obvious that DSB is not lethal in general, indicating that most of induced DSBs can be rejoined or repaired correctly, displaying the spatial distribution of DSBs as major factor in determining lethality [25–27]. Nevertheless, a mechanistic dose-response model has been proposed based on the concept of giant loops, which constitute a level of chromatin organization on a megabase pair length scale [28–30] that suggests DSBs are induced within different loop domains of DNA assumed to be processed independently by cellular repair mechanism. Given giant loop chromatin organization and assumption of two damage classes representing the main point, Giant LOop Binary LEsion (GLOBLE) approach arises as promising model [31]. This model is able to reveal important features of dose-response curves describing cell survival especially transitions from low to high doses in a dose-response correlation.

The effects of combined modality treatments are investigated by using mathematical models to predict cell death as an attempt to fit LQ, MT, and gLQ models to experimental data based on* in vitro* assays, demonstrating that gLQ equation is superior to LQ and MT models in predicting cellular death at high doses of radiotherapy [32]. A significant increasing in biologically equivalent dose may be achieved after addition of radiosensitizing agents to hRT as well as linear accelerators containing new technologies such as flattening filter free (FFF), increases instantaneous dose-rate of X-ray pulses by a factor of 2–6 compared to conventional flattened output [33].

New models for radiobiological cell responses have been proposed and one of those is a simple two-parameter, algorithmic model, which captures the essential biological features of irradiation-induced cell death and associated cell cycle delays. This approach estimates directly the underlying irradiation-induced cell survival and was investigated in mammary carcinoma cell line EMT6/Ro where a comparison of estimated underlying cell survival probability with* in vitro* survival probability data confirms an optimal timing of mixed irradiation/chemotherapy treatments, leading to a development of an accurate spatial and temporal model of tumor progression and cell cycle dynamics [34]. It was already proposed that concurrent chemotherapy with hRT could be beneficial for a number of malignancies taking more variables for survival cell curves and BED calculations [35–37]. Apparently, mathematical models concepts associated with* in vitro* assays and also applying chemotherapy or monoclonal antibodies might be contributed to radiotherapy enhancement.

## 3. Radiation Sensitivity: Targets of Cellular Proliferation Signaling Pathways

A multiplicity of approaches has been investigated in the efforts to enhance ionizing radiation (IR) effects, particularly, signaling cascade involved in cell proliferation. The mitogen-activated protein kinase (MAPK) pathway transduces signals from the cell membrane to the nucleus in response to a variety of different stimuli and participates in various intracellular signaling pathways that control a wide spectrum of cellular processes, including growth, differentiation, and stress responses, and it is known to have a key role in cancer progression [38]. It was already demonstrated in breast cancer cell lines MDAMB-231 that association of SphK1 antagonist FTY720 with IR significantly increased antiproliferative and proapoptotic effects through promoting alterations in MAPK signaling [39]. As is already known Raf-MEK-ERK pathway is a key downstream effector of the Ras small GTPase, the most frequently mutated oncogene in human cancers; thus this signaling network has been subject of intense research and pharmaceutical scrutiny to identify novel target-based approaches for cancer treatment [40]; then small molecule inhibitors of MEK (PD0325901) and Akt (API-2) were subsequently evaluated for their radiosensitizing potential alone and in combination with pancreatic tumor cell lines demonstrating that MEK inhibition results in growth arrest, apoptosis, and radiosensitization of multiple preclinical pancreatic tumor models, and these effects can be enhanced by association with an Akt inhibitor [41].

Since PI3K/AKT/mTOR signaling axis controls cell proliferation and survival this pathway has achieved major importance as a target for cancer therapy [42]. It is already known that activation of these signals is a contributing factor to decreased radiation sensitivity [43], indicating target of mTOR, a downstream kinase of the phosphatidylinositol 3-kinase (PI3K)/AKT survival pathway, may be a target for radiation sensitizing several human cancer cell lines. Its inhibition, currently being proved, increased radiosensitivity of some human cell lines, including SQ20B head and neck carcinoma cells and U251 glioblastoma cells [44]. Radiation sensitivity effect of NVP-BEZ235, a dual PI3K/mTOR inhibitor, reveals enhancement of apoptosis in human glioma cells, as well as cell cycle arrest, resulting in striking tumor radiosensitization, which extended the survival of brain tumor-bearing mice [45–47]. Likewise, NVP-BEZ235 prominently improved the radiosensitivity of PC-3 prostate cancer cells through inducing a G_2_/M arrest and enhanced proapoptotic effect after combined IR [48]. Another study applied the inhibitor RAD001 associated with ionizing radiation in six bladder tumor cell lines: UM-UC3, UM-UC5, UM-UC6, KU7, 253J-BV, and 253-JP, showing arrest in both G_1_ and G_2_ phases of cell cycle when treatments are carried out together, primarily regulated by changes in the levels of cyclin D1, p27, and p21, suggesting that alterations of cell cycle by inhibiting the mTOR signaling pathway in combination with radiation have favorable outcomes and it is a promising therapeutic modality for bladder cancer [49].

The epidermal growth factor receptor (EGFR) is frequently overexpressed in malignant tumors, and its level is correlated with increased cellular radioresistance [50]. One of the defined mechanisms is that EGFR amplification or Ras activation by mutations results in increased clonogenic cell survival and decreased tumor growth delay following irradiation [51]. There is, for instance, a current opinion about therapeutics that target EGFR might enhance the cytotoxic effects of IR. One of these approaches are the humanized monoclonal antibodies, used as anticancer therapy and expected to improve the effectiveness of current therapy to stimulate radiation sensitization; amongst other targets, EGFR blockage (cetuximab), EGFR tyrosine kinase inhibitors (gefitinib), and vascular endothelial growth factor (VEGF) inhibitors such as bevacizumab are still under investigation [52–56]. Cetuximab and IR have shown promising results when performed concomitantly. Previous data have shown that use of monoclonal antibody cetuximab (C225) improves local tumor control after irradiation in FaDu human squamous cell carcinoma (hSCC) due to decreasing repopulation and improving reoxygenation effects as well [57]. Recently, cetuximab was approved for the treatment of patients with recurrent metastatic head and neck squamous cell carcinoma (HNSCC) [58] and phase I/II clinical trials combining bevacizumab with conventional treatments have been performed in advanced/recurrent HNSCC patients. EGFR tyrosine kinase inhibitor (E-TKI) promoted radiosensitization of non-small cell lung cancer (NSCLC) A549 and H3255 cells, with low nitric oxide levels, due to suppression of cell viability when associated with IR [59]. A positive correlation between the presence of a KRAS mutation and radiosensitization after treatment with the EGFR inhibitors erlotinib and cetuximab in several non-small cell lung lineages was lately demonstrated [60]. In addition, it was noticed, for instance, that radiation-induced upregulation of hypoxia-inducible factor-1 alpha (HIF-1*α*) was completely abolished by simultaneous treatment of HNSCC cells with cetuximab [61]. Despite the promising results of applying cetuximab, radiosensitization effect was lost in head and neck tumor cells overexpressing Ras family members such as K-Ras, N-Ras, and H-Ras proteins even as EGFR-independent activation of the RAS/RAF/MEK/MAPK pathway [62–64].

The humanized anti-VEGF monoclonal antibody bevacizumab has single agent activity in previously treated and recurrent cervical, ovarian, and colorectal cancer diseases [65–68], even as tumor sensitivity to adjuvant radiotherapy improvement [69, 70]. The mechanisms of interaction between antiangiogenic agents and IR are complex and involve interactions between tumor cells and tumor microenvironment, including tumor oxygenation, stroma, and vasculature. Radiation resistance of solid tumors toward photon irradiation is caused by attenuation of DNA damage in less oxygenated tumor areas and by increased hypoxia-inducible factor- (HIF-) 1 signaling [71]. When the antiangiogenesis drug Endostar combined with radiotherapy was applied on A549 cells, increased radiation sensitivity by transcriptional factors expression reduction of TGF-*β*_1_ and HIF-1*α* was noticed [72], and it was observed in human colon adenocarcinoma cell line WiDr surviving after radiation therapy by acquiring HIF-1 activity and translocation towards tumor blood vessels in a dependent cellular dynamics after irradiation recurrence, what might suggest basis for targeting HIF-1 after radiation therapy [73, 74], especially in hypoxic tumors.

Interestingly, another promising target to radiosensitize tumors resistant to irradiation is nucleoplasmic calcium. The role of nuclear calcium in tumor cell proliferation was previously determined in HepG2 cells showing decreased proliferation rate under low nuclear Ca^2+^ concentrations due to a mitotic blocking induced by buffering of nuclear Ca^2+^ [75]. Even though the mechanism by which nuclear Ca^2+^ regulates cell proliferation is not completely understood, there are reports demonstrating that activation of tyrosine kinase receptors (RTKs) leads to translocation of RTKs to the nucleus to generate localized nuclear Ca^2+^ signaling which are believed to modulate cell proliferation [76]. We were the first research group who established that nuclear Ca^2+^ buffering decreases EGFR expression, and also the radiosensitization effect of association between nucleoplasmic Ca^2+^ buffering and X-rays in human squamous cell carcinoma A431, preventing ADAM-17 overexpression, induced by IR. Furthermore, this association promoted less tumor cells proliferation and reduced their survival fractions [77], suggesting nucleoplasmic Ca^2+^ as a new target to radiosensitize squamous cell carcinoma.

## 4. DNA Damage Improvements via PARP and DNA-PKcs Inhibitors

The concept of DNA repair centers and the meaning of radiation-induced* foci* in human cells have remained controversial in spite of evidences for formation of these repair centers in a dose-response nonlinearity manner [78]. While IR induces a variety of DNA lesions, including base damage and single strand breaks, DNA double strand break (DSB) is widely considered as the lesion responsible not only for the aimed cell killing of tumor cells, but also for the general genomic instability [79]. As part of an intricate repair complex, poly(ADP-ribose)polymerase 1 (PARP1), functioning as DNA nick-sensor, interacting with base excision repair DNA intermediates containing single strand breaks [80, 81]. Some researchers have shown that PARP inhibitors (PARPi) enhance the cytotoxicity effects of gamma and X-irradiation and alkylating agents, at least when tumor sensitization exceeds effects on normal tissues which could improve clinical outcomes [82, 83]. These inhibitors have gained recent attention due to their unique selectivity in killing tumors with defective DNA repair; therefore, achieving interesting results, by improved radiation sensitivity in UM-SCC1, UM-SCC6, and FaDu cancer cells that, used with cetuximab, decreased nonhomologous end joining (NHEJ) and homologous recombination (HR), mediated DNA double strand break [84]. When olaparib, a potent PARP-1 inhibitor, was investigated in Calu-6 and A549 cells, a human NSCLC, persistent DNA double strand breaks for at least 24 hours after treatment in combination with IR were found, demonstrating radiosensitization to lung cancer cells [85]. Moreover, PARPi was also proposed as a radiosensitizer to Glioblastoma-initiating cells [86, 87] even as another PARP inhibitor ABT-888 (veliparib) enhanced the radiation response of prostate cancer cell lines DU-145 and PC-3 that efficiently promoted abundant senescent cells displaying persistent DNA damage* foci,* and, in human head and neck cancer cells, improved cytotoxicity with ABT-888 and IR was found, compared to either agent alone [88, 89]. Inhibition of histone deacetylases (HDACs) also increases DNA damage, as was noted in A549 lung, DLD-1 colorectal, MiaPaCa2 pancreatic, and UT-SCC15 head and neck squamous cell carcinoma cells, treated with NDACI054 histone deacetylase inhibitor that showed a significant intensification of residual *γ*-H2AX/p53BP1-positive* foci* leading to radiosensitization of these cell lines [90]. In the same way, decay of *γ*-H2AX* foci* correlates with p53 functionality and potentially lethal damage repair in human colorectal carcinoma RKO and prostate cancer DU-145 cells [24, 91]. An alternative treatment strategy to interfere with the proliferative pathways is to apply nimotuzumab, a humanized IgG_1_ monoclonal antibody that specifically targets EGFR in combination with IR [92]. Because of the inhibition of nuclear translocation of EGFR, nimotuzumab and also cetuximab, both antibodies, induce radiosensitization increasing the percentage of dead/dying cells and the yield of *γ*-H2AX* foci* being able to promote intensification of radiosensitivity of malignant cells expressing EGFR and offer potential improving of therapeutic index of radiotherapy [93]. A significant inhibition of radio-induced DNA damage repair, due to inhibited activation of DNA-dependent kinase catalytic subunits (DNA-PKcs) through blocking the PI3K/AKT pathway in A549 cells and MCF-7 breast cancer cells, was observed [92]. In another work, transfected HeLa cells with the anti-DPK3-scFv gene resulted in more sensitivity to IR and diminished DNA repair, which could indicate blockage of DPK3-scFv via targeting DNA-PKcs as a novel biological radiosensitizer for cancer treatment [94].  Table 1 summarizes some molecular targets investigated in their respective tumor cell lines.

## 5. Apoptosis Signaling Pathway Target

Apoptosis is a programmed cell death that is currently of intense research interest in cancer biology and it was already established that expression levels of Bcl-2 family proteins in tumors can modulate apoptosis, influencing tumor behavior and treatment [95], and also, in another way, apoptosis can be triggered intrinsically or extrinsically by DNA damage or other types of severe cellular injures such as reactive oxygen species [96]. For example, Bcl-2 gene has been revealed to be overexpressed in oral cancers predicting outcome in patients treated with definitive radiotherapy [97]. One of the investigated mechanisms of cell death is related with radiation-induced resistance of tumor necrosis factor-related apoptosis-inducing ligand (TRAIL) receptor, an important protein related with failure of recurrent laryngeal cancer. It was suggested that hypermethylation of DR4 CpG island can promote TRAIL radioresistance [98]. Changes in gene expression levels have largely been studied in several cancer types even as their regulatory mechanisms and additionally, microRNAs, a class of endogenous, small noncoding RNAs that negatively regulates gene expression, are considered a new subject of cancer therapy investigation. Recent studies showed that miR-193a-3p was able to radiosensitize both U-251 and HeLa cells by accumulation of intracellular reactive oxygen species increasing DNA damage and also apoptosis directly targeting the antiapoptotic Mcl-1 gene [99], while silencing miR-21 in radioresistant NSCLC A549 cells sensitized them to IR by inhibiting cell proliferation and enhancing cell apoptosis through inhibition of PI3K/Akt signaling pathway [100]. The synergistic effect of resveratrol and IR has been shown in different cancer cell lines effectively acting by enhancing expression of antiproliferative and proapoptotic molecules and inhibiting proproliferative and antiapoptotic molecules, leading to induction of apoptosis through various pathways suggesting resveratrol plus radiotherapy as a therapeutic promise in the near future [101].

Recent investigations have been suggesting autophagy as a cell death pathway that may mediate cancer cells sensitivity to IR even though it could originate a protective mechanism against the treatment itself by removing proteins and organelles that are damaged or, alternatively, produce an effective cell death process [102]. Inhibition of autophagy could sensitize tumor cells to many cytotoxic drugs or reverse resistance to chemotherapeutic drugs, representing a promising strategy to improve the efficacy of cancer treatment [103]. However, the autophagic responses of cancer cells to antineoplastic therapy, including IR, remain a controversial issue.

## 6. Migration and Invasion Pathways Target

Irradiation of primary tumor might promote invasion and favor metastasis by upregulating the expression of genes and activating signaling pathways that are involved in migration and motility. One of the principal pathways involved in alteration of migratory activities is PI3K/Akt signaling pathway that has been implicated in driving metastatic phenotype in thyroid [104], breast [105], and other cancers [106], and PI3K activity is further increased by radiotherapy in certain tumors [107, 108]. Activation of this signaling pathway promotes metastatic transition via stimulation of epithelial-mesenchymal transition (EMT), even as enhancement of migration and invasion [106].

Besides being related to proliferation cascade responses, Akt2, one of the specific isoforms of Akt which is downstream of PI3K, plays important role in promotion of cell migration and invasion. In a xenograft model of colorectal cancer, knockdown of Akt2 in KM20 cell line inhibits liver metastasis; the converse is observed when constitutively active Myr-Akt2 is expressed [109]. It has been noticed that activation of Akt2 increases cell invasion and metastasis of breast and ovarian cancer cells through upregulated integrin signaling [110]; its inactivation also inhibits glioma cell invasion [111] and knockdown of Akt2, rather than Akt1, in the cell line A549 dramatically abolishes its invasive potential [112].

A growing number of studies demonstrate that IR may enhance the migratory and invasive properties of cancer cells via induction of epithelial-mesenchymal transition (EMT). EMT is an embryonic program important for organogenesis in normal development, but its dysfunction can help the survival and dissemination of cancer cells that is characterized by loss of cell-cell contacts, decreased expression of epithelial markers E-cadherin, beta-catenin, and ZO-1, remodeling of the actin cytoskeleton, and increased expression of mesenchymal markers N-cadherin, fibronectin, and vimentin. Several transcription factors have been discovered that can initiate and maintain this process, including Snail, Twist, and Zeb [113]. Furthermore, transforming growth factor beta1 (TGF_*β*_) is a tumor promoter and potent inducer of EMT, though it can be a tumor suppressor during the initial stage of tumorigenesis. Ionizing radiation is able to enhance expression of TGF_*β*_ in various cell lines. This enhancement occurs along with increase of mesenchymal markers and decrease of epithelial markers, as well as alterations in migratory and invasive capabilities of the cells [114, 115]. However, IR is shown to induce TGF_*β*_ activation* in vivo* and sensitize even nonmalignant mammary epithelial cells to undergo TGF_*β*_-mediated EMT [116]. In colorectal cancer IR induces an alteration to a malignant phenotype consistent with EMT* in vitro* [117]. Moreover, TGF_*β*_ is known to be master regulator of EMT, and IR-induced EMT can be reversed by its inhibition; nonetheless other pathways of EMT induction exist as well. Events associated with EMT induced by IR could be reversed through inhibition of TGF_*β*_ signaling with TGF_*β*_R inhibitor SB431542 as was already found in A549 cell line [115]. Another mechanism observed in cervical cancer cells (FIR cells) showed EMT induced by irradiation is dependent on activation of p65 subunit of NF-*κ*B [118]. Pharmacological inhibition of Akt with GSK690693 blocks the expression of ZEB1 and vimentin and restores the expression of E-cadherin following IR, thus preventing the migration and EMT of nasopharyngeal carcinoma cell lines [119] even as knockdown of Akt2 induces reversion of the EMT process in mammary epithelial cell lines [120]. Cell motility on various substrates as well as penetration of membranes is mediated by integrins expressed on the cell surface. Expression of *α*v*β*_3_ in glioma cells [108] and *α*_5_ *β*_1_ in pancreatic cancer [121] is upregulated after IR, facilitating cell migration and invasion. Likewise, integrin *α*_3_ *β*_1_ is overexpressed after IR, promoting the migration of meningioma cells via focal adhesion kinase and extracellular signal-regulated kinase [122]. Moreover, integrin *α*_2_ *β*_1_ is selectively upregulated in irradiated lung cancer cells and is required for aggressive phenotype and invasion in 3D collagen gels and such invasiveness is mediated via PI3K/Akt signaling pathway and then invasion speed* in vitro* can be reduced significantly by PI3K inhibitor LY294002 [123]. Additionally, integrin expression plays role in activation of MMP-2: interaction of MMP-2 with *α*v*β*_3_ integrin is required during its maturation and activation demonstrating localization of active MMP-2 and *α*v*β*_3_ integrin at the migration front accelerates cancer cell migration [124, 125].

Pharmacological inhibition of integrin dependent signaling pathways can be assumed as one of the promising approaches for combined therapy with IR. The function-blocking anti-*α*v*β*_3_ monoclonal antibody 17E6 and *α*v*β*_3_/*α*v*β*_5_ specific antagonist EMD121974 (cilengitide) inhibit* in vitro* matrigel invasion and lung metastasis formation of tumor cells growing in a preirradiated microenvironment [126] and Cilengitide demonstrates strong antimigratory properties in meningioma cells* in vitro*, through combination with IR that allows achieving significant decrease of tumor volumes using intracranial model of human meningioma [127].

**Figure 1:**
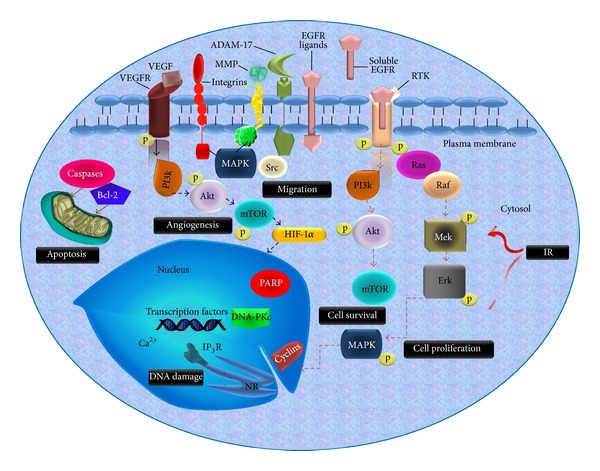
Network of signaling cascade involved in tumor progression and radioresistance. The scheme shows tyrosine kinase axis and its signaling cascade leading to cell proliferation, migration, angiogenesis, apoptosis, and DNA damage, some of them activated by phosphorylation (p). These signaling pathways are involved in radiation resistance and are promising targets to improve radiotherapy (IP_3_R = receptor of inositol 1,4,5-trisphosphate and NR = nucleoplasmic reticulum).

Nonreceptor tyrosine kinase Src is often activated in various types of cancer via mutations or growth factor signaling pathways including insulin-like growth factor-1 receptor (IGFR-1), EGFR, and platelet-derived growth factor receptor (PDGFR). Then, Src plays an important role in focal adhesion disassembly since its expression results in disruption of focal adhesions and stress fibers leading to the loss of adhesion to the extracellular matrix [128]. This Src-mediated disruption of focal adhesions leads to a decrease in cell-cell and cell-ECM adhesion and is an important process central to cell migration and invasion. In addition to its effects on motility, Src may enhance cellular invasion by regulating the expression of MMPs and tissue inhibitors of metalloproteinases [129]. In lung cancer cells EGFR signaling appears to be the dominant mechanism of Src activation. It was already noted that inhibition of Src with submicromolar concentrations of AZD0530 blocks Src and focal adhesion kinase, resulting in significant inhibition of cell migration and matrigel invasion in NSCLC cells [130] suggesting key tyrosine kinase target molecules, combining with IR as a promising radiotherapy enhancement to impair proliferation and invasiveness. Therefore inhibitors of these signaling pathways as part of combination therapy with IR can significantly ameliorate side effect of irradiation.  Figure 1 shows a scheme of cellular signaling pathways related with tumor cell progression.

## 7. Ionizing Radiation and Microenvironment Interface

Enhancement of invasiveness in response to IR can be caused not only by alterations in cancer cell gene expression profile, which increase cell motility and migratory capacities, but also due to modulation of tumor microenvironment. Irradiated tumor microenvironment may exert potential tumor-promoting effects and tumors growing within a previously irradiated bed tend to be more metastatic [126]. Extracellular cell matrix modifications that favor invasiveness are dependent on expression of such enzymes as matrix metalloproteinases (MMPs) MMP-2, MMP-9 and urokinase plasminogen activator (uPA), as well. It is known that expression of these molecules as well as cytokines, which promote invasion in cancer cells, can be induced by IR both in cancer and in stromal cells. Increased levels of MMP-9 and uPA are found in conditioned medium of irradiated neuroblastoma cells [131]. Furthermore, conditioned medium from irradiated nonparenchymal liver cells contains elevated amounts of MMP-2, MMP-9, EGF, and VEGF and promotes invasiveness of sublethally irradiated cultures in hepatoma cell line McA-RH77 [132]. Notwithstanding, proteolytic enzyme urokinase plasminogen activator is upregulated after irradiation in the IOMM-Lee meningioma cells via activation of EGFR, MEK1/2, and p38 signaling pathways, which results in increased tumor invasion and migration* in vitro*. Additionally, inhibitors of these signaling pathways with specific inhibitors AG1478, U0126, and SB203580 show decrease of uPA levels in both basal and irradiated-IOMM-Lee cells [133]. Some proteinases which are expressed by advancing cells of metastatic tumor MMPs are believed to play major role in tumor invasion as they can destroy almost all of basement membrane macromolecules. Injury to the basement membrane can result in the release of proinvasive growth factors which can further stimulate the expression of MMPs [134, 135]. Lewis lung carcinoma cells irradiated in dose of 7.5 Gy demonstrate enhanced expression of MMP-9 and increased invasiveness* in vitro*. When transplanted subcutaneously after subsequent irradiation in xenograft model these cells also have greater lung metastatic potential which is MMP-9 dependent and can be reduced by prototypical MMP-9 inhibitor zoledronic acid [136]. Proteasome inhibitor MG132 potentiates the effect of radiation against the growth and metastasis in NSCLC cells in nontoxic dose. Pretreatment with MG132 followed by irradiation in dose of 4 Gy* in vitro* is shown to suppress cell migration and invasion abilities in A549 and H1299 cancer cell lines, which is accompanied by decreased expression of MMP-2 and MMP-9 in NSCLC cell lines [137]. Irradiation in dose of 5 Gy is shown to induce COX-2 activation in fibroblasts which leads to increased invasiveness of MDAMB-231 cells, cocultivated with these irradiated fibroblasts. This effect is due to PGE2-dependent induction of MMP-2 expression in MDAMB-231 cells and can be completely reversed by COX-2 inhibitor, NS-398 [138].

Pharmacological inhibition of PI3K/Akt signaling pathway has a great therapeutic potential when combined with IR. In thyroid carcinoma cells PI3K inhibitor GDC0941 significantly inhibits lung metastasis in mice bearing irradiated follicular thyroid carcinoma cells. It is of interest that PI3K is not activated in these cells by IR* in vitro*, which means that tumor microenvironment is involved in antimetastatic activities of GDC0941 [139].

Many protocols of irradiation therapy involve irradiation of not only the tumor itself but also nonmalignant cells which surround tumor. As a stress stimulus, irradiation changes significantly gene expression profile of these cells and therefore causes modulation of microenvironment [140]. Enhanced expression of genes associated with proinflammatory response, like COX2 and MMPs, leads to reorganization of extracellular matrix facilitating invasiveness of tumor cells. Inhibition of these signaling pathways in cells of tumor microenvironment can become a promising approach for enhancement of positive effect of radiotherapy.

## 8. Conclusions and Future Perspectives

The increased understanding of the molecular processes underlying cellular sensitivity to IR has led to the identification of novel targets for intervention [87]; combining molecular targeted therapies and radiation may allow for reducing radiation toxicities and improving treatment outcomes [141]. Not only traditional photon therapy but also other modalities applying charged particles can be improved and investigations are being developed, for instance, hadrontherapy, a form of external radiation therapy, which uses beams of charged particles such as carbon ions [71]. Interesting changes at gene level response were achieved in PC-3 prostate cancer cells applying carbon ion irradiation [142] and the hypoxia-induced radioresistance to X-rays can be overcome by carbon-ion beams in SCCVII cell line [143]. Gene therapy also rises as new approach to improve radiosensitivity combining two or more targeting genes. For example, the coexpression of doublecortin (DCX) with secreted protein and rich in cysteine (SPARC) collaboratively diminished radioresistance of glioma cells, interfering with cell cycle turnover and increased irradiation-induced apoptosis [144]. Interestingly, anticancer effects of metformin, the most widely used drug for type II diabetes, alone or in combination with IR, were found radiosensitizing MCF-7 human breast cancer cells and FSaII mouse fibrosarcoma cells by inactivation of mTOR and suppression of its downstream effectors S6K1 and 4EBP1 [145].

The trend of radiation therapy improvements has been focused on tyrosine kinase cascade, the principal signaling pathway to development of new target molecules whose members play a pivotal role on cellular proliferation as migration and tumor invasiveness mediators. Predictable responses might be achieved based on combined IR with specific target molecules able to inhibit overexpressed proteins after radiation exposure. In addition, molecular targeted therapy based on signal transduction pathway alterations detected in cancer offers a tailored treatment possibility including improvements on radiation therapy. Thereby, uses of radiotherapy according to predictive markers would potentially reduce costly over treatment, improve the treatment risk-benefit ratio and cancer outcomes [146], providing further evidence for the importance of intercellular signaling in modulated exposures, where dose gradients are present, and may inform the refinement of established radiobiological models to facilitate the optimization of advanced radiotherapy treatment plans [147]. Furthermore, new models reproducing clinical conditions as closely as possible are needed for radiobiological studies to provide information that can be translated from bench to bedside [148].

Some questions arise concerning how to maximize IR effects combined with these new molecular targets as possible strategies including optimization of dosage and radiation schedule leading to management of toxicities. Further* in vitro* studies are necessary even as systematic reviews focusing on radiobiology and broad molecular targets especially against PI3K/AKT/mTOR pathway that is involved on cellular proliferation as cell migration signaling for the purpose of achieving the best outcome associated with IR to the radiotherapy of the future.

## Figures and Tables

**Table 1:** Molecular targets related with intracellular signaling pathways in different tumor cell lines.

Tumor type	Cell line	Proliferation targets	Migration targets	Angiogenesis targets	DNA targets
Breast	MDAMB-231 MCF-7	SphK1 Akt	PGE2 MMP-2		DNA-PKcs

Head and neck	SQ20B FaDu A431 UM-SCC1 UM-SCC6 UT-SCC15	PI3K/mTOR EGFR ADAM-17 nuclear Ca^2+^		HIF-1*α*	PARP HDAC p53

Glioblastoma	U251	PI3KmTOR	*α*v*β*_3_		

Bladder	UM-UC3 UM-UC5 UM-UC6 KU7 253J-BV 253-JP	mTOR cyclin D1 p27 p21			

Lung	A549 H3255 Calu-6	EGFR E-TKI PI3KAkt	Akt2 TGF_*β*_ *α*_2_ *β*_1_ Src COX-2 MMP-9	VEGFR TGF-*β*_1_	PARP-1 HDAC

Colorectal	WiDr DLD-1 RKO KM20	EGFR PI3KmTOR	Akt2 TGF_*β*_ ST6Gal I	HIF-1*α*	HDAC

Prostate	PC-3 DU-145	PI3KmTOR			PARP-1 p53

Cervix	HeLa FIR	PI3K	p65 NF-kB		DNA-PKcs

Liver	HepG2 McA-RH77 KM20	nuclear Ca^2+^ PI3KAkt	MMP-2 MMP-9	VEGF	
